# Painless Livedoid Vasculopathy in a Patient with G20210A Prothrombin Gene Mutation

**DOI:** 10.1155/2012/910231

**Published:** 2012-09-05

**Authors:** Aibek E. Mirrakhimov, Erwin Velasquez Kho, Alaa Ali

**Affiliations:** Department of Internal Medicine, Saint Joseph Hospital, 2900 N. Lake Shore, Chicago, IL 60657, USA

## Abstract

87 year old Caucasian female with chronic painless non-healing ulcers over malleoli was admitted to the hospital. On a physical examination, there were two bilateral and laterally located malleoli ulcers with no discharge. A thorough work up was done: lower extremities venous and arterial Doppler ultrasound did not show any evidence of venous and arterial disease respectively. Heterozygous G20210A Prothrombin gene mutation was found, and the patient was started on anticoagulation. This case reports highlights a possibility of a painless livedoid vasculopathy presentation in a patient without significant past thrombotic events. Therefore, it is important to consider livedoid vasculopathy in the differential in a patient with painless ulcerative, atrophic and/or nodular skin lesions over the shins and malleoli.

## 1. Introduction

Livedoid vasculopathy (LV) is an underrecognized vascular disorder of the arterial tree, which can be clinically presented with skin ulceration, nodular changes, ankle edema, and plaques with hyperpigmented borders, known as atrophic blanche. Typically, these lesions are painful and are located over the lower shins and ankles [1]. 

The disease pathophysiology is not entirely understood, but thrombosis is believed to play a leading role and inflammation does not seem to contribute to the disease pathogenesis [2–4]. The disease is often incorrectly labeled as vasculitis, whereas, in fact, there is no obvious role of inflammation. Another important issue that the disease has several conflicting names like livedo vasculitis, livedoid vasculitis, and so forth. These factors could contribute to the poor awareness of LV in the general medical practice.

The disease can be missed because of similar presentation in chronic lower extremities venous insufficiency, atherosclerotic peripheral arterial disease, inflammatory vasculitides, pyoderma gangrenosum, and so forth.

LV can be due to secondary causes or due to unknown defect-idiopathic, however, the latter presumption should be made only after a rigorous workup for associated conditions such as autoimmune diseases and procoagulant states. Thus, a thorough diagnostic workup is warranted, and if no alternative etiology is present, a skin biopsy should be considered to confirm LV.

As was mentioned previously, LV is associated with a procoagulant state, favoring clot formation. Prothrombin, which is also known to be II coagulation factor, has a central role in the hemostasis. Mutations in the prothrombin gene are associated with excessive thrombosis, and cardiovascular risk [5], with G20210A mutation is being the most studied. Prothrombin C20209T [6–8] and A19911G [9] genotypes have also been described, however, their role in the procoagulant state is much less clear in comparison to the G20210A mutation. The estimated prevalence of prothrombin gene mutation in the general population is up to 2.6% and up to 8% in patients with a thrombotic event [10].

We report a case of G20210A prothrombin gene mutation and painless LV clinically presenting similar to chronic venous insufficiency of the lower extremities.

## 2. Case Presentation

The patient is an 87-year-old G0P0 Caucasian female which was admitted to the Hospital, because of chronic painless nonhealing skin ulcers over her malleoli bilaterally and shin swelling present for approximately 1 year. Her clinical condition worsened over the last several weeks. The patient denied any fever, chills, shortness of breath, chest pain, cough, abdominal pain, diarrhea, or constipation. The patient does not have past medical history of thrombotic events.

Past medical history is relevant for arterial hypertension, hyperlipidemia, and a resection of a right middle lobe lung mass more than 10 year ago. The patient was using spironolactone, allopurinol, aspirin, carvedilol, rosuvastatin, and pentoxyfilline prior to admission. 

On a physical examination, there were two bilateral and laterally located ulcers over malleoli with a depth of approximately 1 millimeter (mm), height of 1 centimeter (cm), diameter of 0.8 mm, and sharp margins. There was no discharge; the margins of the ulcers were sharp and slightly erythematous without overt induration. Lower venous varicosities and shin swelling were present bilaterally, without local warmth and calf pain. Homan sign was negative bilaterally. Pulses were palpable over arteria dorsalis pedis and arteria femoralis posterior bilaterally. Cardiovascular, chest, abdominal, neurological, HEENT, and eye exams were grossly normal.

Several potential diagnoses were considered: chronic venous insufficiency and lower extremities deep venous thrombosis (DVT) with an emphasis of hypercoagulable state because of a history of pulmonary mass, vasculitis, LV, and skin infection. Complete blood count (CBC), comprehensive metabolic profile (CMP), homocysteine, folic acid, vitamin B12, anticardiolipin antibodies, lupus anticoagulant, factor V Leiden, Protein C activity and Protein C antigen, Protein S activity, D-Dimer, prothrombin mutation, prothrombin time and INR, partial thromboplastin time, blood, and wound cultures were drawn. Relevant diagnostic test results are present in Table 1.

Chest X-ray was done and showed 2.5 cm nodular density over the right hilum and chest CT scan without contrast (the contrast was not given because of compromised renal function present at baseline) showed a 6 mm right cardiophrenic lymph node but did not add additional knowledge regarding the right hilar nodule. However, her past CXR 1 year ago was similar and the recent chest imaging did not find any change from her baseline. The patient denied undergoing pulmonary biopsy and further workup for lung mass at this time.

Lower extremities venous and arterial Doppler ultrasound did not show any evidence of venous and arterial disease, respectively. 

Heterozygous G20210A Prothrombin gene mutation was found (please see the Table 1), and the patient was started on enoxaparin 1 mg/kg once a day and 1 day after warfarin was added to the regimen. The skin biopsy was performed and periodic acid-Schiff staining was used, which showed histopathologic findings characteristic for livedoid vasculopathy, such as deposition of fibrinoid material with the proliferation of endothelial cells with few lymphatic cells. The patient's INR was therapeutic on the fifth hospital day (INR = 2.1) and 1 day after this enoxaparin was discontinued.

On a followup visit after 5 months, the malleoli ulcers were healed, her INR was within the therapeutic range, and the patient did not experience any bleeding or thrombotic episode. 

## 3. Discussion

LV is relatively uncommon disorder, which can be in part explained by nonspecific clinical presentation. Painful skin ulcers and secondary skin changes such as atrophic blanche can be the result of another disorder such as chronic venous insufficiency, vasculitis, pyoderma ganrenosum, and so forth [1]. Some of the conditions associated with LV are presented in Table 2.

The pathophysiology of LV is not clear; however, it is believed that thrombosis has a putative role, whereas inflammation is believed to play a minor role [11]. Several case reports and observations pointed toward a strong association between livedoid vasculopathy and prothrombotic states. These reports will be discussed briefly.

Baccard et al. reported a case of LV in a patient with protein C deficiency, which is a vitamin-K-dependent intrinsic anticoagulant factor [12]. They reported a 65-year-old Caucasian female as in our case without significant history of thrombotic events. Interestingly, heparin, colchicine, aspirin plus dipyridamole therapy failed to improve skin ulceration in this patient, whereas initiation of dapsone was associated with marked improvement of skin lesions. A group from Turkey in a later publication has reported a case of heterozygous protein C deficiency and LV [13]. They reported an 18-year-old man in whom pentoxyfillin in a combination with aspirin and dypiridamole resulted in improved skin lesions. 

Kavala et al. reported a case of 19-year-old male with LV associated with Factor V Leiden [14], which is believed to be the most common inherited procoagulant disorder [15]. Initiation of warfarin improved skin lesions in this patient. Biedermann et al. supported the association between Factor V Leiden and LV, by publishing another case report on this relationship [16].

Tissue plasminogen activator (TPA) is a key intrinsic regulator of clot lysis, and under normal conditions, it is bound to its biological inhibitor, known as plasminogen activator inhibitor (PAI) [17]. Mutations in PAI can increase its affinity to TPA, thus decreasing the availability of TPA under circumstances when clot lysis is physiologically appropriate. Deng et al. reported a case report of PAI 4G/4G homozygosity and LV in a 33-year-old Caucasian female [18]. Warfarin and episodic administration of TPA led to an improvement. Agirbasli et al. found increased stability of PAI in patients with LV [19]. Antunes et al. reported a case of 25-year-old female with PAI 4G/4G homozygosity and prothrombin G20210A mutation [20]. Initiation of TPA has led to resolution of her condition.

A group from Germany has reported a case of 49-year-old female with high homocysteine levels and LV [21]. Commencement of vitamin B12, folic acid, and vitamin B6 supplementation led to a resolution of symptoms. A group from Lebanon has reported a case of 34-year-old female heterozygous for Factor V Leiden, prothrombin G20210A, and C677T methyltetrahydrofolate reductase (MTHFR) homozygosity [22]. C677T genotype of the MTHFR is known to be strongly associated with an increased cardiovascular risk, which is partly explained by prothrombotic effects of homocysteine [22, 23]. Therapy with aspirin, vitamin B12, folic acid, and prednisone led to clinical improvement in their case. 

Several case reports have highlighted the association between G20210A genotype of the prothrombin gene and LV [4, 10, 22, 24–26].

However, in contrast to these reports, our patient had a moderately increased lupus anticoagulant (please see the Table 1), but aPTT, antiphospholipid antibodies, and ANA were negative. Important to mention that the recent study highlighted the possibility of a laboratory error in the measurement of lupus anticoagulant [27], and this might be relevant to our case. 

Another interesting finding in our patient is the absence of LV associated pain. To our knowledge, this is the first case of painless ulcers in LV and this clinical presentation looks particularly intriguing. Our patient did not have diabetes mellitus, her motor function was not impaired and vibratory, light and pain/temperature sensation were preserved, which could potentially explain her clinical presentation. 

Noteworthy mentioning that dermal blood vessels are typically affected and thus, it may explain why arterial Doppler ultrasound was negative, which has a limited sensitivity for small vessels. Several case reports highlight the benefit of anticoagulation therapy, especially in patients with prothrombotic states. Several reports have highlighted the beneficial role for LMWH in the therapy of LV [28, 29]. Our patient was on enoxaparin as well, which was discontinued after a stable therapeutic INR was achieved.

In contrast to the vast majority of published case reports, our patient did not have any significant history of thrombotic events and initially presented with LV in advanced age. This presentation may look somewhat contradictory, since it is clinically expected to have a history of prior thrombotic event at such age with a presumed procoagulant state. This may point toward the possibility that G20210 mutation did not influence the occurrence of LV in our patient. However, based on the prior literature, abnormal coagulation studies in our patient, diagnostic skin biopsy, and a good response to anticoagulant therapy, we think that it is likely that our patient had a procoagulant state-associated LV. 

Overall, this case report highlights a possibility of a painless LV presentation in a patient without significant past thrombotic events. Therefore, it is essential to consider LV in the differential diagnosis even in a patient with painless ulcerative, atrophic, and/or nodular skin lesions over the shins and malleoli. Coagulation studies and autoimmune disease workup should be performed in patients with similar presentation and negative workup for chronic venous insufficiency. Skin biopsy is considered to be a gold standard and should be done to confirm the disease. 

## Figures and Tables

**Table 1:** Results of diagnostic workup.

Lab test (reference range)	Result
Homocysteine (3.7–13.9 Umol/L)	13.7
Anticardiolipin IgG (<23.0 GLP Units)	10.4
Anticardiolipin IgM (<11.0 MPL Units)	1.8
Anticardiolipin IgA (<22.0 APL units)	9.5
ANA	Negative
Factor V leiden	Negative
Antithrombin III activity (>75%)	84
Antithrombin III functionality (218–310 mg/L)	195
Lupus anticoagulant (<1.2)	1.23
Protein C activity (>69%)	69
Protein C antigen (65–140%)	85
Protein S activity (>59%)	65
Prothrombin time (8.9–11.9)	11.4
INR (0.9–1.1)	1.1
C3, C4 and CH50	Within normal limits
Partial thromboplastin time (23–33 seconds)	32
C-ANCA and P-ANCA	Negative
Prothrombin mutation	Heterozygous G20210A prothrombin gene mutation
Blood cultures on two separate occasions	All negative

**Table 2:** Some of the conditions associated with LV.

Autoimmune diseases	Procoagulant states
Systemic lupus erythematosus and antiphospholipid syndrome	Prothrombin G20210A
Rheumatoid arthritis	Factor V leiden
Polyarteritis nodosa	Protein C/S deficiency
Systemic scleroderma	Hyperhomocysteinemia
Mixed connective tissue disease	PAI 4G/4G homozygosity
